# Predicting Professional Identity Among Chinese Nurses Using the Job Demands‐Resources Model: A Comparative Machine‐Learning Study

**DOI:** 10.1155/jonm/8474556

**Published:** 2026-09-29

**Authors:** Jielan Zhong, Qingqing Yang, Jiaqi Xu, Xiangqi Fu, Xiaodi Li, Sheng Wang

**Affiliations:** ^1^ School of Public Health and Nursing, Hangzhou Normal University, Hangzhou, Zhejiang, China, hznu.edu.cn; ^2^ Department of Neurology, The First Affiliated Hospital of Zhejiang Chinese Medical University (Zhejiang Provincial Hospital of Traditional Chinese Medicine), Hangzhou, Zhejiang, China, zjhtcm.com

**Keywords:** career calling, emotion regulation self-efficacy, job demands-resources model, machine learning, professional identity

## Abstract

**Aim:**

To integrate regulatory emotional self‐efficacy and career calling into the Job Demands‐Resources (JD‐R) model and use machine learning to identify model‐derived predictors of nurses’ professional identity.

**Background:**

Nurses’ professional identity is essential for workforce stability, commitment and nursing management. However, limited research has examined professional identity by jointly considering job demands, job resources and personal psychological resources within a predictive modelling framework. Addressing this gap may help identify integrated management priorities for strengthening nurses’ professional identity.

**Methods:**

Convenience sampling with hospital‐specific quotas was used to recruit nurses from five tertiary public hospitals in Zhejiang Province. Data were collected from July to October 2024, yielding 1006 valid responses. The dataset was randomly split into training and testing sets. Boruta feature selection was performed within the training set, and six machine‐learning models were compared using cross‐validation. Model performance was evaluated using MAE, RMSE, *R* ^2^, repeated cross‐validation, bootstrap confidence intervals and a Taylor diagram. SHAP was used to interpret predictor contributions.

**Results:**

Of 23 candidate predictors, 11 were retained. Random forest performed best on the test set (MAE = 8.49, RMSE = 10.97, *R* ^2^ = 0.668; repeated 10‐fold cross‐validated *R* ^2^ = 0.661 ± 0.075). The strongest predictors were career calling, self‐efficacy in managing positive affect, development opportunities and social support. Higher personal and job resources were associated with higher predicted professional identity, whereas job demands were associated with lower predicted values.

**Conclusion:**

Within the integrated JD‐R model and machine‐learning framework, nurses’ professional identity was mainly predicted by personal psychological resources and job resources. These findings are predictive and do not imply causality.

**Implications for Nursing Management:**

Nursing managers may strengthen nurses’ professional identity by enhancing personal psychological resources, improving job resources and addressing job demands, particularly career calling, positive affect self‐efficacy, development opportunities, social support, operational demands, emotional demands and work–family conflict.

## 1. Introduction

Against a backdrop of ever‐increasing demand for healthcare and frequent public health incidents, nursing staff face multiple pressures, including consistently high workloads, occupational risks, strained doctor‐patient relationships and persistent psychological fatigue. A large body of research highlights high levels of burnout, a strong desire to leave the profession, significant mental health issues and a marked decline in professional identity among nurses. Enhancing nurses’ professional identity has become a key factor in maintaining the stability of the nursing workforce [1].

Professional identity reflects an individual’s cognitive, emotional and behavioural attitudes toward their profession. It emphasises the extent to which one’s psychological state is integrated with their work and drives persistent and stable professional behaviour. Research shows that nurses in China generally have a moderate level of professional identity, with some clinical nurses reporting lower levels [2]. A lack of professional identity leads to adverse effects, including impaired mental health, increased turnover, low job satisfaction, reduced quality of care and hindered nursing team development. Nurses’ professional identity plays a fundamental role in their personal growth, psychological well‐being, quality of patient care and long‐term retention in the workforce. Identifying its correlates may inform nursing management and longitudinal research. Multiple factors contribute to how nurses perceive their professional identity. These range from basic demographic variables such as age and gender to individual psychological characteristics, including emotion regulation self‐efficacy and career calling. Workplace conditions also matter considerably; the specific clinical setting, opportunities for professional advancement and availability of collegial support [3–7] all play a part. Most existing studies, however, examine these factors in isolation. Few researchers have attempted to examine multiple influences simultaneously, which points to the need for more integrated approaches. The Job Demands‐Resources (JD‐R) model offers a useful lens for bringing these various factors together [3, 8]^.^ This framework organises workplace elements into two main categories: job demands and job resources [9].

Job demands necessitate sustained physical, intellectual and emotional investment, and excessive or prolonged demands may deplete nurses’ internal and external capacities, thereby diminishing job identification and work engagement. Conversely, job resources, comprising organisational, task‐related and social supports, facilitate goal attainment and buffer the adverse impacts of job demands. Sole reliance on external job resources, however, is insufficient to elucidate nurses’ professional identity fully. Emerging evidence highlights the positive role of nurses’ personal psychological resources in occupational functioning and well‐being [10, 11]^.^. Accordingly, this study focused on career calling and regulatory emotional self‐efficacy as two complementary personal resources within the JD‐R Model. Career calling refers to a sense of purpose and mission that extends beyond self‐interest [12]. For nurses, it reflects the perceived meaning of caring for patients and protecting health, which may help them sustain motivation and strengthen their identification with the profession despite demanding working conditions [13]. However, the relationship between career calling and nurses’ professional identity remains insufficiently examined. Regulatory emotional self‐efficacy refers to individuals’ confidence in managing anger and depressive emotions and in maintaining positive affect [14]. Given the substantial emotional demands of nursing—including exposure to patient suffering and death, distressed family members and challenging interpersonal interactions—this resource may help nurses maintain emotional stability, cope with compassion fatigue and preserve professional well‐being and commitment [15]. Career calling reflects nurses’ sense of meaning and motivation in their professional experience, whereas regulatory emotional self‐efficacy reflects their perceived capacity to manage emotional demands. As these constructs represent distinct aspects of personal resources, both were included as theoretically grounded candidate predictors of professional identity.

Although the JD‐R model has been widely applied in nursing research, previous studies have primarily examined work engagement and burnout, with comparatively limited attention to professional identity as a central outcome integrating both job characteristics and personal psychological resources. Positioning professional identity as the primary outcome extends the JD‐R Model to a relatively enduring construct that is particularly relevant to long‐term workforce stability. Theoretically, professional identity can be conceptualised as a relatively stable, upstream psychological attribute that may shape downstream workforce and care‐related outcomes, including nurse retention, turnover intention, burnout, and quality of care [16]. Unlike work engagement and burnout, which primarily reflect nurses’ current motivational or strain‐related states, professional identity captures a more enduring and internalised sense of belonging to the profession. Practically, strengthening professional identity may provide nursing managers with an actionable target for promoting professional commitment and retention, rather than focusing solely on alleviating burnout after it has developed [17].

Machine learning (ML) refers to computational methods that learn patterns from data and can improve predictive performance as the amount and complexity of available information increase [18]. ML is now widely used in healthcare, economics and other fields [19]. Unlike conventional logistic or linear regression, which generally require prespecified functional forms and focus on the association between individual predictors and an outcome, ML can accommodate multiple predictors simultaneously and is better suited to capturing nonlinear relationships and complex interactions among variables [20]^.^ These characteristics are particularly relevant to professional identity, which is shaped by the interplay of job demands, job resources, personal resources and demographic characteristics. Previous studies have applied ML to examine healthcare professionals’ adaptation to their work environment, professional identity and related outcomes [21]. Given its ability to evaluate multivariable predictive patterns and compare out‐of‐sample performance, the present study employed several ML algorithms to develop a predictive model of nurses’ professional identity. Boruta was used for feature selection, and SHapley Additive exPlanations (SHAP) were applied to interpret the relative contribution of each predictor. These analyses were intended to identify and rank predictive variables rather than to establish causal effects.

This study aimed to use the JD‐R model as a theoretical framework, incorporating job demands, job resources, career calling and emotion regulation self‐efficacy alongside demographic factors into the analysis, and to use ML methods to identify the variables that best explain and predict professional identity. Based on this, six different models were tested to predict professional identity scores. Furthermore, the researchers employed the SHAP method to assess variable importance in the predictive models [22]. The findings may inform the design of management strategies to support nurses’ professional identity.

## 2. Methods

### 2.1. Research Design and Participants

This single‐province, multicentre cross‐sectional study employed convenience sampling with hospital‐specific quotas. The sampling frame consisted of registered clinical nurses working in five tertiary public hospitals in Zhejiang Province, China. These hospitals were selected based on the feasibility of study coordination and their willingness to assist with participant recruitment. Between July and October 2024, eligible nurses at each hospital were invited to participate voluntarily until a target quota of approximately 200 valid responses was reached. The study did not attempt to include all eligible nurses at each hospital. Eligible participants were registered nurses actively engaged in clinical practice. Trainee nurses, nonclinical staff, administrative and logistics personnel and nurses on extended leave were excluded. The nursing departments assisted only in distributing the study invitation and questionnaire link. Because hospital‐level invitation logs were not retained, the exact number of nurses who received the invitation could not be determined; therefore, a conventional response rate based on the number invited could not be calculated. Data were collected online using Wenjuanxing. Upon opening the link, nurses were presented with an electronic study information and informed consent page. They could access the questionnaire only after actively confirming that they had read the study information and agreed to participate. No data were collected from nurses who did not provide consent. Quality control measures included limiting submissions to one per device, requiring completion of all items and screening submitted questionnaires for invalid responses. A total of 1039 questionnaires were submitted. After excluding 33 questionnaires completed in less than one minute, 1006 valid questionnaires were retained, yielding a valid questionnaire rate of 96.82%. The study was reported in accordance with the STROBE guidelines. With the completed checklist and additional methodological details provided in Supporting Information S3.

### 2.2. Sample Size Calculation

The sample size was informed by Vapnik–Chervonenkis dimension theory [23]. This approach was selected because the primary objective was to develop and evaluate multivariable prediction models for a continuous outcome. Conventional power analysis is primarily designed to test a prespecified hypothesis and requires assumptions about the expected effect size, statistical test, significance level and desired power [24]. These assumptions did not directly correspond to the objective of comparing the out‐of‐sample predictive performance of multiple ML algorithms. Events‐per‐variable criteria were also not directly applicable because professional identity was analysed as a continuous outcome rather than as a binary or time‐to‐event outcome. VC theory links the complexity of a hypothesis class to its expected generalisation error and remains a useful reference framework for evaluating the generalisation behaviour of predictive models [25]^.^ However, because the exact VC dimension varies across algorithms, the calculation was treated as a supplementary theoretical assessment of sample adequacy rather than an exact model‐specific sample size requirement. In this approach, the sample size equals the number of selected features plus one. The expected generalisation error was set at 10%, and the confidence parameter *δ* was set to 0.05. General demographic data, job resources, job demands, emotion regulation self‐efficacy and career calling serve as independent variables; the number of features included and professional identity serve as the dependent variable to be predicted. Ultimately, the VC dimensions comprise 30 dimensions, with a generalisation error of 10% (0.1). According to the formula *N* ≥ (1/*ϵ*)(*d*_*V* *C*_ln(1/*ϵ*) + ln(1/*δ*)), at least 721 participants were required to meet the theoretical threshold. Given a 20% invalid questionnaire rate, the target sample size was 902. In addition, as a routine cross‐check, based on the empirical rule of requiring a sample size 5–10 times the number of questionnaire items, a 93‐item questionnaire would require approximately 465–930 participants. The final valid sample included 1006 participants. These participants were randomly allocated to a training set of 753 participants and a testing set of 253 participants. The training sample therefore exceeded the VC dimension‐based theoretical benchmark of 721 observations.

### 2.3. Measurement

#### 2.3.1. General Demographic Data

Based on the research objectives and content, and following a thorough review, collation and synthesis of the literature, the researcher and research team designed a questionnaire to collect general demographic data on clinical nurses. This section comprises 12 items, primarily covering gender, age, educational background, professional title, average monthly income, marital status, establishment status, years of service, children, night‐shift frequency, department and commuting time.

#### 2.3.2. Job Resources Scale

Job resources were assessed using the 15‐item Job Resources Scale developed by Li Jienan et al. The scale was selected because it operationalises the job resources component of the internationally established JD‐R model through four dimensions relevant to healthcare practice: work autonomy, social support, feedback and development opportunities [9]. Items are rated on a 5‐point Likert scale, with higher scores indicating greater perceived job resources. The scale has previously been applied in studies of Chinese healthcare professionals, supporting its contextual relevance to the present study [26]^.^ In the present sample, the scale demonstrated excellent internal consistency, with a Cronbach’s α of 0.918 and a McDonald’s ω of 0.921 for the total scale. Subscale Cronbach’s α coefficients ranged from 0.920 to 0.942. Confirmatory factor analysis (CFA) using MLR estimation indicated an acceptable overall fit for the four‐factor structure: *χ* ^2^/df = 5.51, comparative fit index (CFI) = 0.952, Tucker–Lewis index (TLI) = 0.940, root mean square error of approximation (RMSEA) = 0.067, 90% CI [0.062, 0.072] and standardised root mean square residual (SRMR) = 0.038. Standardised factor loadings ranged from 0.82 to 0.94.

#### 2.3.3. Job Demands Scale

Job demands were assessed using the 11‐item Job Demands Scale developed by [27]. The scale was selected because its three dimensions, operational demands, emotional demands and work–family conflict, correspond closely to the physical, emotional and interpersonal demands encountered in nursing practice. Items are rated on a 5‐point Likert scale, with higher scores indicating greater perceived job demands. The scale has previously been applied among Chinese healthcare workers, supporting its contextual relevance to the present sample [28]. In the present sample, the scale demonstrated excellent internal consistency, with a Cronbach’s α of 0.929 and a McDonald’s ω of 0.932 for the total scale. Subscale Cronbach’s α coefficients ranged from 0.877 to 0.959. CFA was conducted using the robust maximum likelihood estimator (MLR), and model fit was evaluated using the CFI, TLI, RMSEA and SRMR: *χ* ^2^/df = 7.21, CFI = 0.962, TLI = 0.949, RMSEA = 0.079, 90% CI [0.071, 0.086] and SRMR = 0.045. Standardised factor loadings ranged from 0.72 to 0.95.

#### 2.3.4. Career Calling Scale

Career calling was assessed using the 12‐item Calling Scale originally developed and validated by Dobrow and Tosti‐Kharas [29] and subsequently adapted and validated for Chinese populations by Pei and Zhao [30]. The scale was selected because both the original and Chinese versions have demonstrated acceptable psychometric properties. It has also been applied among Chinese nurses to examine the relationships between career calling, organisational commitment and work engagement, supporting its contextual relevance to nursing research [31]. The scale has a unidimensional structure and uses a 5‐point Likert response format, with higher scores indicating a stronger sense of career calling. In the present sample, the scale demonstrated excellent internal consistency, with both Cronbach’s α and McDonald’s ω equal to 0.930. CFA using MLR estimation indicated an acceptable overall fit for the unidimensional structure: *χ* ^2^/df = 6.94, CFI = 0.952, TLI = 0.941, RMSEA = 0.077, 90% CI [0.070, 0.084] and SRMR = 0.030. Standardised factor loadings ranged from 0.68 to 0.78.

#### 2.3.5. Regulatory Emotional Self‐Efficacy Scale

Regulatory emotional self‐efficacy was assessed using the 12‐item Regulatory Emotional Self‐Efficacy Scale developed and validated by Caprara et al. [14] across samples from Italy, the United States and Bolivia. The scale was subsequently adapted for Chinese populations by Weng et al. [32]. It was selected because it is an internationally validated instrument that assesses self‐efficacy in managing positive affect, anger and depressive emotions, all of which are relevant to the emotional labour involved in nursing practice [33, 34]. The scale comprises three dimensions: self‐efficacy in managing positive affect, self‐efficacy in managing anger and self‐efficacy in managing depressive emotions. Items are rated on a 5‐point Likert scale, with higher scores indicating greater confidence in regulating emotional experiences. In the present sample, the scale demonstrated excellent internal consistency, with both Cronbach’s α and McDonald’s ω equal to 0.923 for the total scale. Subscale Cronbach’s α coefficients were 0.945 for self‐efficacy in managing positive affect, 0.892 for self‐efficacy in managing anger and 0.936 for self‐efficacy in managing depressive emotions. CFA using MLR estimation indicated a good fit for the three‐factor structure: *χ* ^2^/df = 4.46, CFI = 0.965, TLI = 0.955, RMSEA = 0.059, 90% CI [0.053, 0.065] and SRMR = 0.040. Standardised factor loadings ranged from 0.83 to 0.93.

#### 2.3.6. Nurses’ Professional Identity Scale

Professional identity was assessed using the 30‐item Nurses’ Professional Identity Scale developed by Liu et al. [35]. The scale was specifically designed to capture the multidimensional nature of professional identity among Chinese nurses. It was selected because of its nursing‐specific content and established application in Chinese nursing research [36]. The scale comprises five dimensions: professional self‐concept, professional support, professional social skills, coping with professional setbacks and professional self‐reflection. Items are rated on a 5‐point Likert scale, with higher scores indicating stronger professional identity. In the present sample, the scale demonstrated excellent internal consistency, with a Cronbach’s α of 0.966 and a McDonald’s ω of 0.967 for the total scale. Subscale Cronbach’s α coefficients ranged from 0.904 to 0.956. CFA using MLR estimation indicated an acceptable overall fit for the five‐factor structure: *χ* ^2^/df = 4.40, CFI = 0.927, TLI = 0.920, RMSEA = 0.058, 90% CI [0.056, 0.060] and SRMR = 0.040. Standardised factor loadings ranged from 0.76 to 0.92.

### 2.4. Data Collection

Data were collected online from July to October 2024. The researchers explained the study’s objectives, methods and procedures to the nursing departments of each hospital and obtained their consent. Before completing the questionnaire, participants were informed of the study’s necessity and methodology; it was clearly stated that the data would be used solely for research purposes; instructions for completing the questionnaire were provided; and participants were given the option to participate in the survey voluntarily. Furthermore, participants were informed that they could withdraw at any time without suffering any adverse consequences.

### 2.5. Data Analysis

#### 2.5.1. Statistical Analysis

All analyses were conducted in *R* 4.5.2 using a fixed random seed (20240705). Data processing and modelling used the tidymodels ecosystem, including parsnip, recipes, rsample, workflows, tune and yardstick [37]. The model engines included rpart, glmnet, kernlab, xgboost and ranger; feature selection used Boruta. SHAP values were computed with fastshap and visualised with shapviz; and psychometric analyses used psych and lavaan. Continuous variables were summarised as means and standard deviations, whereas categorical variables were summarised as frequencies and percentages. Between‐group differences in professional identity were examined using one‐way analysis of variance, independent‐samples *t*‐tests, or chi‐square tests, with a two‐sided *p* < 0.05 considered statistically significant. Twenty‐three candidate predictors, comprising 12 demographic and occupational variables and 11 JD‐R dimension scores, were considered for predicting the continuous professional identity score.

The dataset contained no missing values; therefore, no imputation was performed. Before any data‐dependent processing, the sample was randomly divided into training (75%) and test (25%) sets, stratified by quartiles of the professional identity score. Categorical candidate predictors were treated as factors during Boruta selection. None was retained in the final feature set; therefore, dummy coding was not required in the primary model. Numeric predictors were standardised to zero mean and unit variance using parameters estimated from the training data. Within each cross‐validation resample, the preprocessing recipe was estimated using the analysis fold only and then applied to the corresponding assessment fold. After model selection, the final recipe was fitted to the complete training set and applied unchanged to the held‐out test set [37]. All preprocessing was training‐based: categorical candidates were treated as factors for Boruta, while numeric predictors were z‐standardised within each cross‐validation fold and the final recipe was applied unchanged to the test set.

#### 2.5.2. Predictive Modelling

Feature selection was performed exclusively within the training set using the Boruta algorithm [38]. Boruta is a random forest (RF)‐based, all‐relevant wrapper method that compares the importance of each original predictor with that of randomly permuted shadow features. Predictors are retained only when their importance consistently and significantly exceeds the maximum importance achieved by the shadow features [38, 39]. Boruta was selected because the study aimed to retain all predictors containing potentially relevant information about professional identity rather than derive only a minimal predictor subset. Furthermore, it does not require a prespecified linear functional form and can identify predictors involved in potentially nonlinear relationships and complex interactions. The held‐out test set was not used during feature selection or model tuning.

Six regression algorithms were compared: decision tree (DT), linear model (LM), elastic net (ENet), support vector machine (SVM), extreme gradient boosting (XGBoost) and RF. Comparing these algorithms enabled a systematic assessment of different linear and nonlinear modelling approaches. Each algorithm was implemented as a workflow combining the preprocessing recipe and model specification. Except for the LM, hyperparameters for each model were tuned within the training set using stratified 10‐fold cross‐validation and a space‐filling grid of 20 candidate configurations. The selected configurations were refitted using the complete training set. Model performance was evaluated on the held‐out test set using the mean absolute error (MAE), RMSE and coefficient of determination (*R* ^2^). Ninety‐five percent confidence intervals were estimated using 2000 bootstrap resamples of the test set. Model stability was additionally assessed using repeated 10‐fold cross‐validation with five repeats. The models were further compared using a Taylor diagram [40, 41], which based on the law of cosines, simultaneously represents the correlation coefficient (*R*), the standard deviation (*σ*) and the centred RMSE (CRMSE) in a single polar plot, enabling a comprehensive visual comparison of model performance in terms of pattern similarity, error magnitude and variability, the best‐performing model was retained as the final model. SHAP values were estimated using fastshap with 50 Monte Carlo simulations and visualised with shapviz. These values quantify each predictor’s contribution to model predictions rather than causal effects [42]. The selected predictors were mapped onto the job‐demand, job‐resource and personal‐resource components of the JD‐R model, enabling their relative importance and contributions to predicted professional identity to be compared within this theoretical framework.

#### 2.5.3. Reliability, Validity and Common Method Bias

Because the dimension scores of the five instruments were used as candidate predictors in the ML models, their psychometric properties were re‐examined in the present sample. Internal consistency was assessed with Cronbach’s α and McDonald’s ω; both exceeded 0.87 for all total scales and subscales (Cronbach’s *α* = 0.877–0.966; McDonald’s *ω* = 0.882–0.967), indicating excellent reliability. Construct validity was evaluated by CFA using robust maximum‐likelihood estimation [43]. The a priori factor structure of each multidimensional instrument fitted the data acceptably to well, including the three‐factor structure of job demands, the four‐factor structure of job resources, the three‐factor structure of regulatory emotional self‐efficacy, the five‐factor structure of professional identity (*χ* ^2^/df: 4.40–7.21, CFI: 0.927–0.965, TLI: 0.920–0.962, RMSEA: 0.058–0.079 with 90% CI, SRMR: 0.030–0.045) and all standardised factor loadings were ≥ 0.68, supporting the hypothesised dimensionality.

Because all constructs were self‐reported, common method bias was examined using Harman’s single‐factor test [44], an unrotated principal component analysis of all 80 items extracted 16 components with eigenvalues > 1, and the first component accounted for 32.83% of the total variance, well below the 40% threshold, indicating that common method bias did not substantially distort the results [45]. Full reliability coefficients and fit indices for each scale are reported in Supporting Information Table S1.

### 2.6. Ethics

This study adheres to ethical principles. It has been approved by the Institutional Review Board of the researcher’s university and by an external body (Ethics Review Number: 2024043). Informed consent has been obtained, participants’ privacy is protected and data will be used solely for research purposes and kept strictly confidential.

## 3. Results

### 3.1. Descriptive Statistics

This analysis ultimately included 1006 participants. To verify the model’s generalisation ability, the data were randomly split into training (75%; *n* = 753) and test (25%; *n* = 253) sets. In the overall sample, there were 961 females (95.5%) and 45 males (4.5%), with the age group highly concentrated between 31 and 40 years old. Statistical tests showed no significant differences in general baseline characteristics between the training and test sets. Further general demographic data are presented in Supporting Information Table S2.

### 3.2. Boruta Feature Selection and Extraction

This study considered 23 candidate predictors and used the Boruta algorithm, applied within the training set, to screen them. The Boruta algorithm calculates the importance of each variable based on an RF and establishes a random benchmark by constructing shadow features. Shadow variables are generated by randomly substituting the original variables and are used to assess whether the importance of a true variable is significantly higher than that of random noise. Green indicates confirmed features (Confirmed), whose importance is significantly higher than the upper bound of the shadow variable’s importance (shadowMax); red indicates features that have been rejected, meaning their significance is lower than the random baseline; yellow indicates features whose significance is currently uncertain (Tentative); and blue indicates shadow features, which are used as random reference variables for importance comparisons. Boruta retained 11 of the 23 candidate predictors above the shadowMax threshold for subsequent analyses. These included operational demands, emotional demands, work–family conflict, work autonomy, social support, feedback, development opportunities, career calling and self‐efficacy in managing positive affect, depressive emotions and anger. Any general demographic variables with a lower importance score than the shadow variables were excluded. See Figure 1 for details of the variables.

**FIGURE 1:**
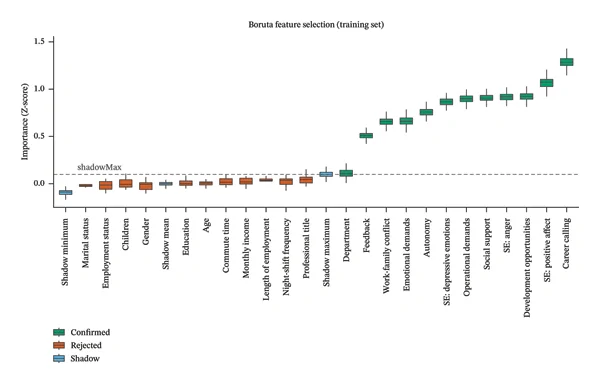
Results of the Boruta algorithm. Of the 23 candidate predictors, 12 demographic variables (red) were rejected by Boruta, while the remaining 11 dimension‐level predictors (green) were confirmed. “SE” is explicitly defined as “self‐efficacy”.

### 3.3. Six Predictive Models and Interpretability

Eleven key feature variables were selected and extracted to predict professional identity. Six common ML models were compared: DT, LM, ENet, SVM, XGBoost and RF. To evaluate the predictive performance of each model, the MAE, RMSE and *R* ^2^ were calculated for a comprehensive comparison. The smaller the MAE and RMSE, the smaller the discrepancy between the predicted and actual values, and the higher the model’s accuracy, the higher the *R* ^2^ value (which ranges from 0 to 1), the better the model’s fit. The RF achieved the best performance across all three metrics (MAE = 8.49, RMSE = 10.97, *R* ^2^ = 0.668), followed closely by XGBoost (*R* ^2^ = 0.666), both clearly outperforming the SVM (*R* ^2^ = 0.637), ENet and LM (*R* ^2^ = 0.545) and the DT, which showed the largest errors and lowest *R* ^2^ (0.484). Bootstrap 95% confidence intervals and repeated 10‐fold cross‐validation with five repeats provided additional evidence of internal model stability. The Taylor diagram also placed the RF closest to the observed reference point, with the highest correlation and lowest centred RMSE among the models. Based on the above comparison, this study ultimately selected the RF model as the base model for the subsequent feature importance analysis. The performance results for each model are shown in Table 1 and Figure 2.

**TABLE 1:** Predictive performance of the six machine‐learning models for nurses’ professional identity.

Model	*R* ^2^ (95% CI)	RMSE (95% CI)	MAE (95% CI)	Cross‐validated *R* ^2^ (mean ± SD)	SD ratio
Random forest	0.668 (0.577–0.748)	10.973 (9.787–12.225)	8.487 (7.630–9.340)	0.661 ± 0.075	0.763
XGBoost	0.666 (0.577–0.745)	10.991 (9.789–12.193)	8.533 (7.691–9.376)	0.656 ± 0.074	0.759
Support vector machine	0.637 (0.545–0.715)	11.346 (10.184–12.499)	8.886 (8.054–9.725)	0.614 ± 0.076	0.806
Elastic net	0.545 (0.431–0.649)	12.704 (11.279–14.259)	9.788 (8.827–10.788)	0.584 ± 0.087	0.765
Linear model	0.545 (0.431–0.649)	12.714 (11.280–14.282)	9.781 (8.817–10.779)	0.583 ± 0.087	0.769
Decision tree	0.484 (0.339–0.625)	14.236 (11.865–16.939)	9.888 (8.726–11.190)	0.514 ± 0.104	0.927

*Note:* *R* ^2^ = coefficient of determination (squared Pearson correlation); SD ratio = ratio of predicted to observed standard deviation. The random forest (bold) was the best‐performing model and was carried forward for SHAP interpretation. Random forest and XGBoost outperformed the support vector machine, elastic net, linear model and decision tree.

Abbreviations: MAE = mean absolute error, RMSE = root mean square error.

**FIGURE 2:**
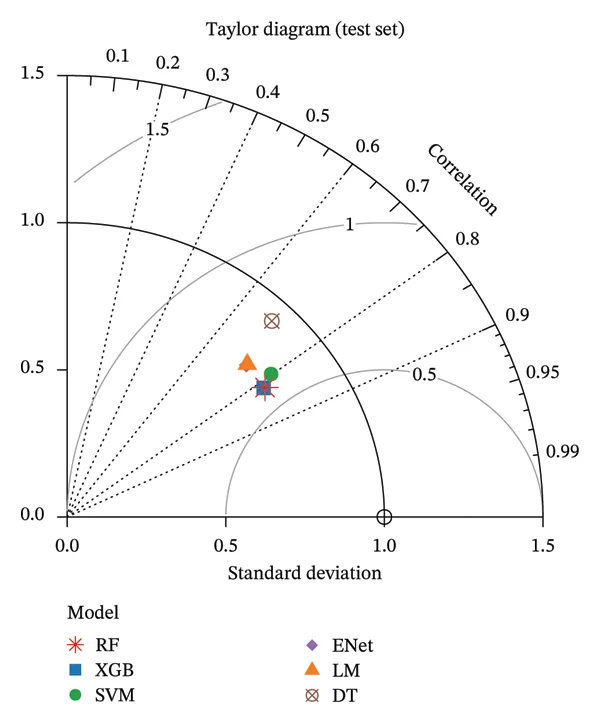
Results of the Taylor diagram. Among all the models compared, the random forest (RF) model performed best. Its corresponding data points are closest to the observed reference point in the Taylor plot, with a relatively high correlation coefficient and a low centering RMSE, resulting in better overall prediction accuracy than other models.

### 3.4. Predictive Modelling and Interpretability

Mean absolute SHAP values were used to quantify the average magnitude of each predictor’s contribution to the RF predictions. Career calling was the highest‐ranked predictor (SHAP = 3.19), followed by self‐efficacy in managing positive affect (SHAP = 3.00), development opportunities (SHAP = 1.86), social support (SHAP = 1.85) and self‐efficacy in managing anger (SHAP = 1.75). Work autonomy, operational demands, self‐efficacy in managing depressive emotions, work–family conflict and emotional demands made smaller contributions and feedback had the lowest mean absolute SHAP value (SHAP = 0.54).

Figure 3 presents the predictors in descending order of their mean absolute SHAP values. Figure 4 shows the distribution and direction of their contributions to predicted professional identity. Each point represents one observation, and its colour indicates the feature value, with red representing higher values and blue representing lower values. The *x*‐axis represents the SHAP contribution relative to the model baseline. Higher personal and job resource values were generally associated with higher predicted professional identity, whereas higher job demands were associated with lower predicted values.

**FIGURE 3:**
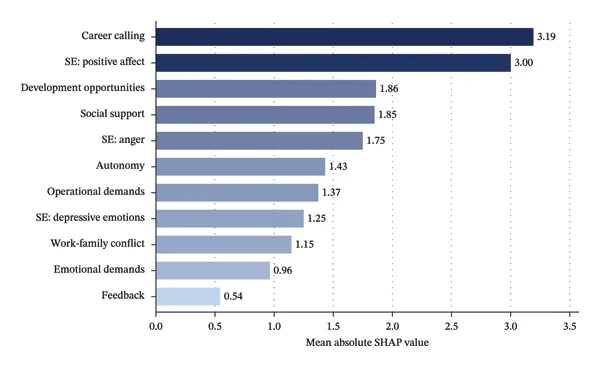
Mean absolute SHAP values of the 11 predictors in the random forest model. Larger values indicate greater average contributions to model predictions, and darker bars indicate higher‐ranked predictors. Predictors are ordered from the largest to the smallest mean absolute SHAP value. “SE” denotes self‐efficacy.

**FIGURE 4:**
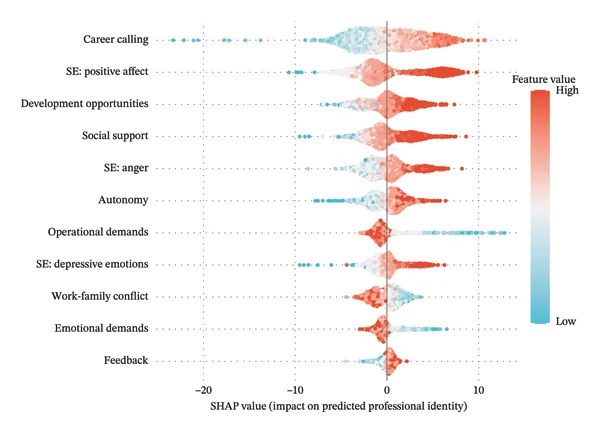
SHAP beeswarm plot showing the distribution and direction of the contributions of 11 predictors to model‐predicted professional identity. Each point represents one observation, and colour indicates the feature value (red = high and blue = low). Higher feature values concentrated in the positive SHAP region were associated with higher predicted professional identity, whereas those concentrated in the negative region were associated with lower predicted values. These patterns reflect associations with model predictions rather than causal effects. “SE” is explicitly defined as “self‐efficacy”.

## 4. Discussion

Guided by the JD‐R Model, this study compared six ML models incorporating job demands, job resources and personal resources to predict professional identity among Chinese nurses. The RF model showed the best predictive performance, and SHAP ranked 11 model‐derived predictors. These comprised demands (operational demands, emotional demands and work–family conflict), job resources (work autonomy, social support, feedback and development opportunities) and personal resources (career calling and the three dimensions of regulatory emotional self‐efficacy). Career calling and self‐efficacy in managing positive affect were the strongest predictors, followed by development opportunities and social support. The observed pattern was consistent with the motivational and health‐impairment pathways proposed by the JD‐R model: higher personal and job resources were associated with higher predicted professional identity [46, 47]^,^ whereas higher job demands were associated with lower predicted values [48]. By incorporating career calling and regulatory emotional self‐efficacy as personal resources and positioning professional identity as the focal outcome, this study broadens the application of the JD‐R Model beyond commonly studied outcomes such as work engagement and burnout. However, these theory‐consistent predictive associations do not establish the temporal or causal mechanisms underlying either pathway.

Career calling was the highest‐ranked model‐derived predictor of nurses’ professional identity (SHAP = 3.19). The SHAP beeswarm plot showed that higher career calling values were generally associated with higher predicted professional identity. This pattern is consistent with previous research linking career calling to professional enthusiasm, maturity and higher professional identity [49]. According to the JD‐R model, resources serve to stimulate individuals’ intrinsic motivation and promote work engagement. As a powerful intrinsic psychological resource, a career calling represents a nurse’s inner sense of mission and strong identification with the nursing profession; it is a deep‐seated motivation that arises from within, viewing the nursing profession as a life purpose and a means of realising one’s values. A career calling helps nurses integrate their nursing work deeply with their personal values, fostering a profound sense of identification with the profession. Nurses with a strong sense of career calling tend to demonstrate greater vitality, dedication and focus; their sustained commitment to their work enables them to affirm the value of nursing psychologically, thereby reinforcing their professional identity [50].

The SHAP beeswarm plot showed that higher values across all three dimensions of regulatory emotional self‐efficacy were generally associated with positive SHAP values and higher predicted professional identity. Self‐efficacy in managing positive affect ranked second overall and first among the three dimensions (SHAP = 3.00), followed by self‐efficacy in managing anger (SHAP = 1.75; fifth overall) and depressive emotions (SHAP = 1.25; eighth overall). This ranking indicates greater predictive relevance for confidence in sustaining positive affect than for regulating negative emotions. The overall pattern is consistent with previous cross‐sectional evidence linking regulatory emotional self‐efficacy to professional identity [51, 52]. Within the JD‐R Model, regulatory emotional self‐efficacy can be conceptualised as a personal resource theoretically relevant to both the motivational and health‐impairment processes. Self‐efficacy in managing positive affect reflects confidence in sustaining positive emotions and has been associated with emotional competence, work engagement and resilience, which may help contextualise its high predictive ranking [53–55]. In comparison, self‐efficacy in managing anger and depressive emotions may be relevant to nurses’ responses to emotional demands, nurse–patient conflict and negative work experiences, consistent with their smaller predictive contributions [56–58]. Thus, the three dimensions may reflect personal resources relevant to different aspects of the JD‐R motivational and health‐impairment processes.

The SHAP beeswarm plot further shows that, for all three dimensions of emotion regulation self‐efficacy, high feature values, shown as red points, were predominantly concentrated in the positive SHAP value region, indicating that greater emotion regulation self‐efficacy was consistently associated with higher predicted professional identity. The three dimensions nonetheless differed markedly in the magnitude of their contributions. Self‐efficacy in managing positive affect (SHAP = 3.00) was the second most important predictor overall, and was by far the strongest emotion regulation dimension. In contrast, self‐efficacy in managing anger (SHAP = 1.75) ranked fifth, while self‐efficacy in managing depressive emotions (SHAP = 1.25) ranked eighth, indicating progressively smaller contributions. This suggests that, for professional identity, the capacity to actively generate and sustain positive emotions may be more central than the down‐regulation of negative emotions. The relationship between emotion and professional identity in nursing has been well documented in cross‐sectional research, and the present study reaffirms that nurses’ emotion regulation self‐efficacy was associated with professional identity.

According to the JD‐R model, self‐efficacy in managing positive affect—as an internal resource—refers to a nurse’s belief in their ability to actively elicit and sustain positive emotional experiences and to cultivate positive emotions in nursing practice to counteract emotional exhaustion at work; it thus plays a significant role in the formation and consolidation of nurses’ professional identity. Positive emotional experiences in nursing, such as successful care delivery, recognition from patients, colleagues or superiors and the refinement of nursing skills, all serve to reinforce professional identity. Self‐efficacy in managing positive affect enhances nurses’ ability to regulate emotions and cultivate self‐belief [53] and belief in one’s emotional competence is a vital component of professional self‐identity. Nurses who are confident in their ability to generate and sustain positive emotions are better equipped to cope with the emotional labour inherent in patient care. As a psychological resource, self‐efficacy in managing positive affect helps build positive nurse–patient relationships and provides external positive feedback that reinforces professional identity; it enhances emotional resilience, maintains mental health and safeguards the continuity of professional identity [54, 55]. Encouraging nurses to proactively create and sustain positive experiences therefore provides an important emotional foundation for strengthening their identification with professional values, internalising psychological resources and consolidating them into a stable sense of professional identity [59–61]. Self‐efficacy in managing anger ranked fifth and was associated with higher predicted professional identity, possibly reflecting more adaptive responses to nurse–patient conflict and role‐related frustration [58]. Self‐efficacy in managing depressive emotions ranked eighth and showed the smallest contribution among the three dimensions, with a modest association that may reflect better recovery from negative experiences [56, 57].

In addition to the two personal resources of career calling and self‐efficacy in managing positive affect, the job resources of development opportunities and social support were also important predictors of professional identity. Development opportunities and social support ranked third and fourth among all predictors, respectively. Consistent with previous studies, a significant positive correlation was found between social support and professional identity, and social support can directly promote the formation of professional identity [62]. Within the JD‐R Model, job resources are positioned within the proposed motivational pathway and are theoretically associated with work motivation and professional identity. Social support refers to the emotional and practical support nurses receive from their team, managers and organisation in the course of their work; it is a key job resource associated with nurses’ professional identity. As a profession characterised by high emotional investment and high stress levels, support from superiors, assistance from colleagues and understanding from patients not only provides nurses with the instrumental resources needed to carry out their nursing duties but also conveys an emotional resonance that acknowledges the value of the nursing profession. This reinforces nurses’ sense of meaning and belonging in their nursing roles, thereby consolidating their professional identity [24]. Development opportunities provide nurses with avenues for continuous learning and career progression, enabling them to perceive the ongoing growth of their professional judgment, whereas work autonomy reflects nurses’ discretion to make decisions based on their professional judgement. The continuous accumulation of growth and the sense of involvement in autonomous decision‐making integrate the nurse and the nursing profession into a single entity that grows together, thereby enhancing identification with the nursing role and upholding professional identity [63, 64]. However, among the 11 significant variables, feedback made the smallest contribution to professional identity. This may be because feedback is largely regarded as an operational‐level resource aimed at adjusting work performance and thus has limited explanatory power for deeper psychological constructs such as professional identity [65, 66].

The SHAP beeswarm plot showed that higher operational demands, emotional demands and work–family conflict were generally associated with negative SHAP values and lower predicted professional identity. Among the three job demands, operational demands showed the largest predictive contribution (SHAP = 1.37; seventh overall), followed by work–family conflict (SHAP = 1.15; ninth overall) and emotional demands (SHAP = 0.96; tenth overall). Thus, all three dimensions displayed a consistent negative predictive pattern, although their contributions differed in magnitude. Within the JD‐R framework, these variables represent job demands, and their negative predictive patterns are consistent with the proposed health‐impairment pathway. This pathway theoretically relates sustained job demands to psychological strain and resource depletion. Operational demands may be associated with fatigue and a reduced sense of meaning in routine nursing work, whereas emotional demands have been linked to emotional exhaustion arising from the regulation of emotions during difficult care situations [66, 67]. Work–family conflict reflects tension between professional and family responsibilities and may coincide with lower professional belonging when job demands interfere with personal life [68].

Overall, the RF model identified career calling and self‐efficacy in managing positive affect as the most informative predictors of nurses’ professional identity. These findings indicate several potential priorities for nursing management. Nursing managers could consider values‐based induction, mentorship, professional recognition and transparent career‐development pathways to help nurses connect their work with professional meaning [69, 70]. Emotion regulation and mindfulness training, together with structured support following critical incidents, could be considered to support nurses’ confidence in managing positive affect and anger [71, 72]. Supportive leadership, team‐based peer support and continuing education may also represent relevant approaches to strengthening social support and development opportunities [73, 74]. In addition, workload monitoring, emotional support and flexible or family‐friendly scheduling could be considered in response to operational demands, emotional demands and work–family conflict, which were associated with lower predicted levels of professional identity [75, 76]. Although some of these strategies are supported by previous evidence, their effectiveness in strengthening nurses’ professional identity cannot be inferred from the present predictive analysis and requires further evaluation in longitudinal and intervention studies.

### 4.1. Limitations

This single‐province, multicentre cross‐sectional study employed convenience sampling with hospital‐specific quotas and included nurses from five tertiary public hospitals in Zhejiang Province, China. Consequently, the sample may not be representative of nurses working in secondary, community, or private hospitals or in other geographical regions, which limits the generalizability of the findings. Because hospital identifiers were not retained, between‐hospital differences could not be assessed, and hospital‐stratified or leave‐one‐hospital‐out validation was not feasible. Accordingly, the reported predictive performance represents internal validation within the same source population rather than evidence of external generalisability. External validation across different institutions, hospital levels, geographical regions and time periods is required before the model can be applied to broader nursing populations. The cross‐sectional design precludes causal inference, and SHAP results describe associations with model predictions rather than causal effects. Other potentially relevant personal resources, such as resilience and psychological capital, and physiological factors were not measured and warrant future study.

Beyond this, the analysis was confined to psychological and working constructs, with no physiological variables, such as fatigue or drowsiness, taken into account. As a result, the potential influence of physical symptoms on professional identity warrants further investigation. It should also be noted that the relatively low *R* ^2^ value suggests that other unmeasured variables may have contributed to the variance in professional identity.

Although RF models outperform traditional linear regression in handling nonlinear relationships, they lack intuitive parameter coefficients. This study employed the Boruta algorithm for feature selection; however, in psychometric data, such as emotion regulation self‐efficacy and career calling, multicollinearity across scale dimensions may have led to conceptual overlap among selected features. Although post hoc explanations were provided using SHAP scores, these merely describe the model and do not enable path validation based on theoretical assumptions.

In view of these limitations, future research could incorporate additional factors that may influence nurses’ professional identity or refine the model’s structure, ideally drawing on face‐to‐face interviews, mixed‐methods strategies and longitudinal designs to yield deeper and more temporally informed insights into these relationships.

## 5. Conclusions

By integrating the JD‐R model with ML, encompassing RF modelling and SHAP analysis, this study identified career calling, self‐efficacy in managing positive affect, development opportunities and social support as the most important predictors of professional identity among nurses; higher values of these personal and job resources were associated with higher predicted professional identity. The results offer hospital managers a concrete evidence base for refining management strategies and developing proactive, targeted interventions.

## Funding

The authors received no specific funding for this work.

## Conflicts of Interest

The authors declare no conflicts of interest.

## Supporting Information

Additional supporting information can be found online in the Supporting Information section.

## Supporting information


**Supporting Information** This study includes the following Supporting Information. Full details of reliability, validity and common method bias analyses are reported in Table S1. The demographic characteristics of the participating nurses are summarised in Table S2. In accordance with the STROBE guidelines, the completed reporting checklist is provided in Supporting Information S3.

## Data Availability

The data that support the findings of this study are available from the corresponding author upon reasonable request.
